# Catastrophic acetabular wear following polyethylene liner dissociation with locking-tab damage after total hip arthroplasty: a case report

**DOI:** 10.3389/fsurg.2026.1979979

**Published:** 2026-10-23

**Authors:** Weida Li, Zhixing Liang, Zhuangzhuang Dong, Qian Sun, Yu Hou

**Affiliations:** Department of Joint Surgery, Affiliated Hospital of Hebei University, Baoding, China

**Keywords:** catastrophic acetabular wear, locking-tab damage, metallosis, polyethylene liner dissociation, revision total hip arthroplasty, total hip arthroplasty

## Abstract

**Background:**

Polyethylene liner dissociation is an uncommon but potentially severe complication of total hip arthroplasty (THA). Loss of the intended bearing interface can permit direct femoral-head–acetabular-shell articulation, with progressive shell wear, metallic debris deposition, and acetabular bone loss.

**Case presentation:**

A 73-year-old woman developed left hip pain after an accidental sprain in February 2025, having reported no hip-related symptoms after primary THA in 2020. The index Mindray implants included a 48-mm acetabular shell, a highly cross-linked polyethylene liner, a 28-mm femoral head, and a size-10 stem. Radiographs and CT with three-dimensional volume-rendered reconstruction were used to assess acetabular bone loss, classified as Paprosky type IIA. Revision revealed polyethylene liner dissociation, catastrophic shell wear-through, extensive black metallic debris deposition, and a well-fixed femoral stem. Partial locking-tab damage was identified on gross inspection, but formal retrieval analysis was not performed. Acetabular revision with iliac-crest autografting was performed while retaining the femoral stem. At 6 months, the Harris Hip Score improved from 46 to 82, and radiographs showed a stable acetabular component with evidence of graft incorporation.

**Conclusion:**

This case illustrates the potential for clinically occult progression from liner dissociation to catastrophic head-on-shell wear. Gross locking-tab damage did not establish the initiating failure mechanism. Component-preserving acetabular revision was associated with a favorable short-term clinical and radiographic outcome in this patient.

## Introduction

Polyethylene liner dissociation is an uncommon but potentially severe complication of modular total hip arthroplasty (THA). Loss of the intended bearing interface permits direct contact between the femoral head and acetabular shell, potentially resulting in progressive shell wear, metallic debris generation, and damage to surrounding tissues (1, 2). Imaging can identify abnormal femoral-head position and help locate a dissociated liner (3). Napier et al. reported eight dissociations among 4,751 THAs using Pinnacle polyethylene liners (0.17%), illustrating the rarity of the event in that implant system (4).

The present report describes advanced polyethylene liner dissociation with catastrophic acetabular wear and grossly visible partial locking-tab damage. Its clinical value lies in the discrepancy between the absence of reported prodromal symptoms and the extent of structural damage, together with the technical challenges of acetabular reconstruction while retaining a well-fixed femoral stem. The initiating failure mechanism could not be established because formal retrieval analysis was not performed.

## Case presentation

### Preoperative information

A 73-year-old woman underwent primary left THA at a local hospital in 2020 through a posterolateral approach. According to the available operative records, the index implants were manufactured by Mindray and included a 48-mm acetabular shell, a highly cross-linked polyethylene liner, a 28-mm femoral head, and a size-10 femoral stem. Immediate postoperative radiographs from the index THA were unavailable; therefore, the original acetabular component orientation could not be reliably reconstructed.

At revision, the patient was 161 cm tall and weighed 66 kg, corresponding to a body mass index of 25.5 kg/m^2^. Her medical history included hypertension for approximately 8 years, and her American Society of Anesthesiologists physical status classification was II.

The patient reported no hip-related pain, subjective limb shortening, clicking, squeaking, or clunking before February 2025. She then developed left hip pain following an accidental sprain. The pain was aggravated by activity and partly relieved by rest, with progressive limitation of walking.

On admission, physical examination revealed a healed surgical scar on the left hip, no obvious deformity or swelling, and normal local skin temperature. Tenderness was noted at the midpoint of the inguinal region and near the greater trochanter, with a positive axial percussion test. Left hip range of motion (ROM) was flexion 5–60°, restricted extension, abduction 0–30°, adduction 0–10°, internal rotation 0–15°, and external rotation 0–20°. The Patrick (FABER) test was positive.

### Preoperative assessment and surgical planning

Laboratory evaluation showed a white blood cell count of 4.98 × 10⁹/L, C-reactive protein of 2.14 mg/L, and erythrocyte sedimentation rate of 25 mm/h. These serum findings did not strongly suggest an active infectious process. No preoperative joint aspiration was performed, and no intraoperative microbiological cultures or histopathological specimens were subsequently obtained. Low-grade periprosthetic joint infection (PJI) therefore could not be definitively excluded (5).

Preoperative anteroposterior pelvic radiographs and computed tomography (CT), including three-dimensional volume-rendered CT reconstruction (CT-VR), were obtained to assess the acetabular component, the pattern of bone loss, and its relationship to the surrounding pelvic structures (Figure 1). The acetabular rim was largely intact, the anterior and posterior columns were preserved, and the medial wall remained intact without substantial destruction. The bone loss was predominantly cavitary, without a major segmental defect. No transacetabular fracture line or separation between the superior and inferior hemipelvis was identified. Together with the intraoperative findings, these features were most consistent with a Paprosky type IIA acetabular defect. No abnormal motion between pelvic segments was observed intraoperatively, and pelvic discontinuity was confirmed to be absent. Revision was planned to reconstruct the failed acetabular side, with femoral component retention dependent on intraoperative assessment of fixation and taper condition. The clinical course is summarized in Table 1.

**Figure 1:**
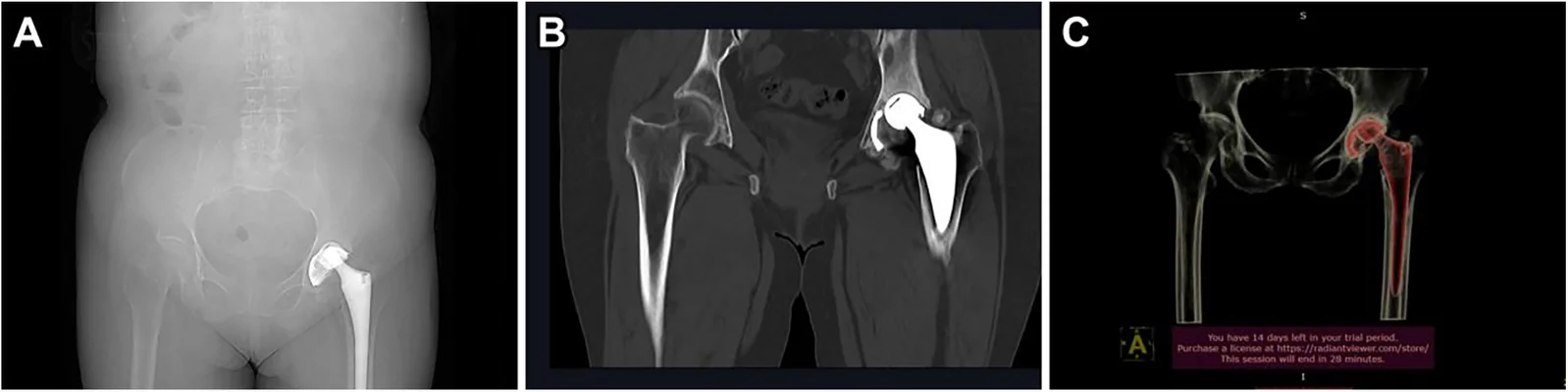
Preoperative imaging. **(A)** Anteroposterior pelvic radiograph. **(B)** Representative preoperative CT image demonstrating the acetabular bone-loss pattern. **(C)** Three-dimensional volume-rendered CT reconstruction showing the components and surrounding pelvic bone.

**Table 1:** Clinical timeline.

Time or phase	Clinical course
2020	Primary left THA through a posterolateral approach using Mindray components.
2020–February 2025	No reported hip-related pain, subjective shortening, or mechanical noises.
February 2025	Sprain followed by left hip pain and walking limitation.
Pre-revision assessment	Radiographs and CT with volume-rendered reconstruction; acetabular defect classified as Paprosky IIA.
Revision surgery	Liner dissociation, shell wear-through, metallic debris, and partial locking-tab damage; acetabular reconstruction with retention of the well-fixed stem.
Postoperative day 1	Bedside rehabilitation, ankle pumps, and quadriceps/gluteal isometric exercises initiated.
Postoperative day 3	Walker-assisted ambulation with weight bearing as tolerated.
6 months	Harris Hip Score 82 (preoperative 46); painless functional motion; stable cup and radiographic graft incorporation.

### Overview of the revision strategy

Revision surgery was performed through the previous posterolateral approach. Exploration revealed polyethylene liner dissociation, catastrophic wear-through of the acetabular shell by the femoral head, and extensive black metallic debris deposition. The femoral stem remained well fixed and was retained. Acetabular reconstruction comprised removal of the damaged shell, autologous iliac-crest bone grafting, and implantation of a new acetabular component.

### Detailed surgical procedure

Dense fibrotic and heavily metal-stained tissues surrounded the acetabulum. Standard electrocautery was less effective in these tissues, and careful sharp dissection was required for exposure and debridement. Extensive black metallic debris deposition consistent with metallosis was observed in the periacetabular tissues (Figure 2A).

**Figure 2:**
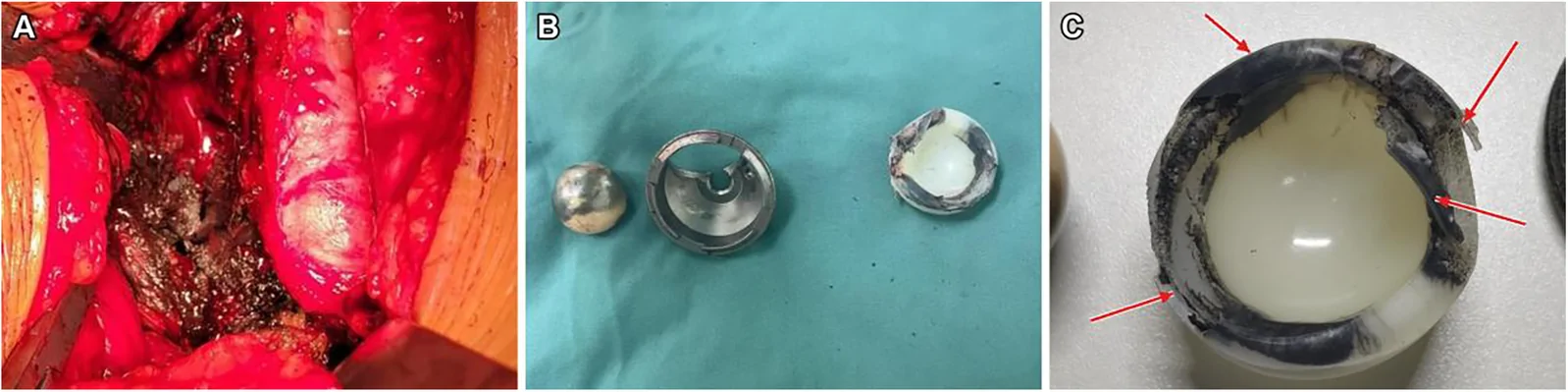
Intraoperative and retrieved-component findings. **(A)** Marked black tissue staining and debris in the operative field. **(B)** Retrieved femoral head, damaged metallic acetabular shell, and polyethylene liner, from left to right. **(C)** Cropped close-up of the retrieved polyethylene liner. Arrows indicate areas of suspected rim damage requiring correlation with direct inspection of the locking features. Gross appearance alone cannot establish the sequence of damage or the initiating failure mechanism.

The retained femoral stem restricted acetabular exposure, requiring controlled bone-hook traction on the prosthetic neck. The firmly attached shell was initially released circumferentially with an osteotome. After exposure of the rim, a Gigli saw was used to cut the posterior portion of the damaged cup and facilitate removal (Figure 2B). After removal, the acetabular defect was confirmed to be a contained cavitary defect with preservation of the acetabular rim, medial wall, and both anterior and posterior columns. Approximately 10–15 mL of morselized cancellous autograft was used to fill and augment the cavitary defect. The graft was intended to restore local bone stock and did not serve as a load-bearing structural graft. Local autograft was insufficient because no native femoral head was available in this revision setting, and the quantity and quality of cancellous bone obtainable locally after debridement and reaming were limited. Cancellous autograft was therefore harvested from the ipsilateral iliac crest. Although the patient was 73 years old, her general condition and iliac bone quality were considered adequate for harvesting the modest graft volume required.

Gross inspection of the retrieved polyethylene liner demonstrated partial damage to the locking tabs. However, no formal retrieval analysis of the locking tabs, locking groove, or failure surfaces was performed. Gross inspection alone could not determine whether the observed tab damage represented a primary locking-interface failure contributing to liner dissociation or secondary damage sustained after dissociation during prolonged abnormal articulation. Therefore, the initiating mechanism of liner dissociation could not be established with certainty.

After removal of the original femoral head, the retained trunnion was inspected. No gross corrosion, fretting, deformation, or other visible taper damage was identified, supporting retention of the well-fixed femoral stem.

Following bone grafting, acetabular preparation initially targeted approximately 45° of inclination and 15° of anteversion. Because of the available host-bone geometry and limited superior support, a lower final inclination was accepted to maximize host-bone coverage and component support.

A 48-mm cementless modular acetabular cup (AK Medical) with a 40-mm internal shell diameter was implanted using press-fit fixation. Host-bone contact was achieved primarily in DeLee–Charnley zones II and III, with partial contact in zone I. Initial mechanical stability was supplemented with two screws placed into the superior iliac bone. Primary cup stability was thus obtained from preserved host-bone contact, press-fit fixation, and supplementary screw fixation rather than from the bone graft.

A 40/32-mm highly cross-linked polyethylene (HXLPE) liner (AK-L-II, Cat. No. 2329–4032, AK Medical) with a reported polyethylene thickness of 4 mm was implanted. The liner was compatible with the acetabular shell and was secured through a circumferential locking mechanism with anti-rotational features. A 32-mm ceramic femoral head was selected instead of the previous 28-mm head to increase the head–neck ratio, jump distance, and impingement-free range of motion. No titanium adapter sleeve was used.

A dual-mobility articulation was considered but was not selected because the indication for revision was not recurrent instability. The patient had no history of recurrent dislocation, the abductor mechanism and periarticular soft tissues were preserved, and satisfactory stability was obtained during intraoperative trial reduction. A conventional fixed-bearing HXLPE construct was therefore selected.

Trial reduction demonstrated satisfactory stability and soft-tissue tension. The replacement ceramic head showed satisfactory seating on the retained taper during intraoperative assessment. The retained stem was manufactured by Mindray and the replacement head was supplied by AK Medical; formal manufacturer documentation confirming cross-manufacturer taper compatibility was unavailable. Manual intraoperative assessment after implantation demonstrated approximately 110° of flexion and 20° of abduction. These angles were visual estimates during surgery.

The total operative time was 125 min, with an estimated blood loss of 1,100 mL. Given the significant blood loss, an intraoperative component blood transfusion was promptly administered, comprising 4 units of type A Rh-positive packed red blood cells (PRBCs) and 400 mL of matched fresh frozen plasma (FFP). The transfusion proceeded smoothly, and the patient's vital signs remained stable throughout the procedure, with no transfusion-related complications such as hemolytic, febrile, or allergic reactions. Following accurate instrument and sponge counts and thorough wound irrigation, the incision was closed in layers, and a single closed-suction drain was placed.

### Postoperative course

Postoperative rehabilitation began on day 1 with bedside mobilization, ankle-pump exercises, and isometric contractions of the quadriceps and gluteal muscles. On postoperative day 3, the patient began walker-assisted ambulation under a weight-bearing-as-tolerated protocol. The phased rehabilitation program implemented for this patient is summarized in Table 2.

**Table 2:** Phased postoperative rehabilitation program implemented for this patient.

Time after revision	Rehabilitation activities	Goals
Days 0–3	Ankle-pump exercises and isometric quadriceps and gluteal contractions; turning and sitting up in bed, with bedside rehabilitation beginning on day 1. Walker-assisted standing and ambulation began on day 3, with weight bearing as tolerated as prescribed.	Prevent venous thrombosis and restore basic mobility.
Weeks 1–6	Walking with a walker or two crutches, with a gradual increase in walking distance; hip and knee range-of-motion exercises; gentle quadriceps and gluteal exercises.	Restore basic range of motion and gait.
Weeks 6–12	Gradual reduction in walking-aid use according to radiographic and clinical findings; progressive hip abductor and extensor exercises; balance and stair training.	Restore a normal gait pattern and muscle strength.
After 12 weeks	Progressive resistance training, stationary cycling, swimming, and other low-impact activities.	Restore activities of daily living and long-term function.

Given the use of a ceramic head on a previously used cross-manufacturer trunnion without a titanium adapter sleeve, the patient was informed of the potential implant-related risks, including ceramic-head fracture and taper-related fretting or corrosion. She was advised to seek medical attention promptly if new-onset hip pain, clicking, squeaking, grinding, instability, or sudden deterioration in function developed.

A dedicated surveillance strategy was established. Standard anteroposterior pelvic and lateral hip radiographs are planned at approximately 6 and 12 months after revision and annually thereafter if the patient remains asymptomatic, to assess component position, fixation, osteolysis, radiolucent lines, and evidence of ceramic or modular-junction failure. If new pain, mechanical symptoms, or abnormal radiographic findings occur, further investigations will be performed as clinically indicated. CT may be used to assess occult ceramic fracture, component position, osteolysis, or other structural abnormalities. Serum metal-ion testing and metal-artifact-reduction MRI may be considered if taper-related corrosion or an adverse local tissue reaction is suspected.

Postoperative acetabular inclination measured on the anteroposterior pelvic radiograph was approximately 27°, whereas anteversion measured on postoperative CT was approximately 11°. Figure 3 shows the postoperative frontal image. These measurements reflected the final reconstruction after adjustment for the available host-bone support.

**Figure 3:**
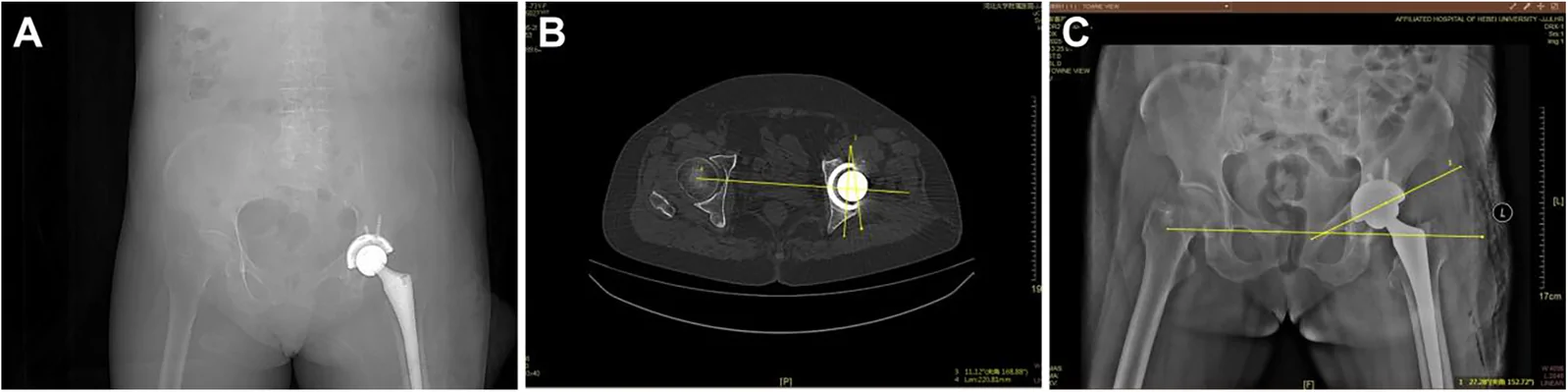
Post-revision imaging. **(A)** Anteroposterior pelvic radiograph showing the reconstructed left acetabular component; inclination was approximately 27°. **(B)** Postoperative CT image used for assessment of acetabular component anteversion, measured at approximately 11°. **(C)** AP pelvic radiograph with the inclination measurement.

At the 6-month follow-up, the Harris Hip Score had improved from 46 preoperatively to 82. Clinical assessment demonstrated approximately 110° of hip flexion and 20° of abduction with painless functional motion; these were approximate clinical estimates rather than standardized goniometric measurements. The patient walked without a limp, and no clinically apparent leg-length discrepancy or reported postoperative complication was identified. Formal radiographic leg-length measurement was not performed. The patient reported substantial relief of hip pain, improved walking ability, and satisfaction with the postoperative recovery.

Radiographs at 6 months showed a stable acetabular component, without obvious migration or progressive radiolucent lines compared with the immediate postoperative images. The grafted region demonstrated bridging trabeculae across the graft–host bone interface, early remodeling, and no obvious graft resorption, consistent with radiographic graft incorporation.

## Discussion

This case illustrates an advanced manifestation of polyethylene liner dissociation: catastrophic shell wear-through accompanied by metallic debris deposition, acetabular bone loss, and partial locking-tab damage. The absence of reported prodromal symptoms despite extensive structural damage is the principal clinical observation. Liner dissociation and acetabular revision with stem retention have previously been described; the contribution of this report is the potentially silent progression and the technical decisions required during reconstruction (1, 2, 6).

### Possible mechanisms and clinically occult progression

Polyethylene liner dissociation may involve incomplete seating, impingement, edge loading, repetitive mechanical stress, implant design, and damage to the locking interface. In standardized lever-out tests, Beckmann et al. found reduced resistance to dissociation after removal of anti-rotation tabs in the tested Pinnacle construct (7). Perkins et al. described liner deformation, rim impingement, and frequent anti-rotation-tab shearing in 26 retrieved dissociated Pinnacle liners (8). These findings support examining the locking interface, but their application to the Mindray construct remains uncertain. In this patient, gross tab damage could have preceded dissociation or developed secondarily during abnormal articulation. Without formal retrieval analysis or immediate postoperative imaging from the index THA, the failure sequence and contribution of original cup malposition cannot be determined.

Clinical presentation varies substantially. Cheppalli et al. reported early postoperative dissociation accompanied by mechanical noises, whereas catastrophic acetabular wear has also been reported after years of apparently satisfactory function (2, 9). Burger and Nowakowski described severe eccentric cup abrasion after polyethylene wear, including a potentially prolonged asymptomatic phase (10). In the present case, the sprain may have unmasked or aggravated an existing process. The extensive wear at revision suggests preceding abnormal articulation, but the history cannot establish its onset or duration. The date of symptom onset should therefore not be treated as the date of structural failure.

### Consequences of prolonged head-on-shell articulation

Loss of the polyethylene bearing permits direct femoral-head–acetabular-shell articulation and progressive component damage. Reports of catastrophic wear and ceramic-head penetration through metallic cups provide clinical parallels for the severe shell damage observed here (2, 11). Continued abnormal contact can generate metallic debris and coexist with tissue fibrosis and acetabular bone loss. Gross black staining in this patient was consistent with metallosis; however, serum cobalt/chromium measurements, metal artifact reduction sequence MRI, and histopathological assessment, including assessment for aseptic lymphocyte-dominated vasculitis-associated lesion, were not performed. The local tissue response and any systemic metal exposure therefore could not be comprehensively characterized.

### Revision decision-making and technical considerations

Catastrophic shell wear-through required acetabular component replacement. This differs from dissociation with an intact, securely fixed shell and undamaged locking grooves, in which shell retention has been described (4, 9). The well-fixed femoral stem was retained to avoid extraction-related femoral bone loss and fracture risk. Kaku et al. reported stem retention during acetabular revision after bipolar hip arthroplasty, supporting the feasibility of component preservation in selected patients, although that clinical setting differs from the present case (6).

Preserving the stem also restricted exposure. Bone-hook traction, sharp dissection of fibrotic tissues, circumferential shell release, and Gigli-saw-assisted extraction were needed before iliac-crest graft reconstruction. Severely worn cup edges can create additional handling hazards during revision (10). The operative duration of 125 min, estimated blood loss of 1,100 mL, and transfusion requirement indicate a technically demanding procedure. This case provides no comparative evidence that stem retention reduced operative time or blood loss.

The final cup orientation represented a patient-specific compromise rather than an ideal target. The relatively low inclination of approximately 27° was accepted to maximize host-bone coverage and support in the setting of the available acetabular bone stock. However, a low cup inclination may increase the risk of anterior impingement and posterior edge loading, with potential implications for polyethylene wear. This consideration is particularly relevant given the relatively thin polyethylene liner used in this reconstruction. Continued radiographic surveillance is therefore warranted.

The head–neck reconstruction used in this case requires particular caution. The retained Mindray femoral stem and the replacement AK Medical ceramic head were supplied by different manufacturers, and no titanium adapter sleeve was used. Although no gross corrosion, fretting, deformation, or visible taper damage was identified intraoperatively, visual inspection and satisfactory seating do not establish taper compatibility and cannot exclude microscopic taper damage. Potential concerns include taper mismatch, fretting, corrosion, stress concentration, and ceramic-head fracture. Use of a cross-manufacturer ceramic head on a previously used trunnion without a titanium adapter sleeve should not be emulated. This construct should not be interpreted as a recommended or reproducible revision strategy; manufacturer-confirmed taper compatibility and appropriate revision components are required when planning head–neck reconstruction. The favorable 6-month result does not establish the long-term safety of this head–neck combination.

Bearing dimensions also need to be interpreted within the specific implant system. Wu et al. found no increase in liner-related complications when larger heads were paired with the smallest compatible cups in a primary-THA cohort using highly cross-linked polyethylene (12). Those findings do not validate the 4-mm revision liner or the head–stem pairing used here. Long-term results reported for a specific contemporary cup and cross-linked polyethylene bearing by Bedard et al. likewise cannot be extrapolated to this revision construct (13). The present outcome is limited to improved function and stable radiographic findings at 6 months.

### Limitations

This single case and 6-month follow-up cannot establish the initiating mechanism, comparative effectiveness of the revision strategy, or long-term implant survival. Formal retrieval analysis and immediate postoperative imaging from the index THA were unavailable, limiting reconstruction of the original failure sequence and acetabular component orientation. Pelvic discontinuity was absent on preoperative imaging and intraoperative assessment. Post-revision inclination was measured on an anteroposterior pelvic radiograph and anteversion on postoperative CT; these are measurements obtained using different imaging modalities.

The absence of aspiration, intraoperative cultures, and histology prevented definitive exclusion of low-grade PJI (5). Metal-ion testing, metal artifact reduction sequence MRI, and histopathological characterization of the metal-related tissue response were also unavailable. Cross-manufacturer taper compatibility was not formally confirmed, and a ceramic head was used without a titanium adapter sleeve; the short follow-up cannot establish the safety of this combination. Intraoperative and 6-month ROM values of approximately 110° of flexion and 20° of abduction were clinical estimates rather than standardized goniometric measurements. Leg length was assessed clinically without formal radiographic measurement. Radiographic graft incorporation at this interval does not establish complete osseointegration or durable fixation.

## Conclusion

This case demonstrates catastrophic acetabular wear following polyethylene liner dissociation, with partial locking-tab damage identified on gross inspection. The initiating mechanism could not be established. Severe wear despite an absence of reported prodromal symptoms suggests clinically occult progression, with the sprain potentially revealing an existing process. Acetabular revision with retention of the well-fixed femoral stem was associated with improved function and stable radiographic findings at 6 months. Longer follow-up is required to assess the durability of the reconstruction.

## Data Availability

The original contributions presented in the study are included in the article. Further inquiries can be directed to the corresponding author.
